# Antimicrobial activity, cytotoxicity and chemical analysis of lemongrass essential oil (*Cymbopogon flexuosus*) and pure citral

**DOI:** 10.1007/s00253-016-7807-y

**Published:** 2016-08-26

**Authors:** Emmanuel C. Adukwu, Melissa Bowles, Valerie Edwards-Jones, Heather Bone

**Affiliations:** 1 Faculty of Applied Sciences, University of the West of England, Coldharbour Lane, Bristol, BS16 1QY UK; 2 Manchester Metropolitan University, Manchester, UK

**Keywords:** *Acinetobacter baumannii*, Lemongrass oil, Multi-drug resistance (MDR), Toxicity, IC_50_

## Abstract

The aim of this study was to determine the antimicrobial effects of lemongrass essential oil (*C. flexuosus*) and to determine cytotoxic effects of both test compounds on human dermal fibroblasts. Antimicrobial susceptibility screening was carried out using the disk diffusion method. Antimicrobial resistance was observed in four of five *Acinetobacter baumannii* strains with two strains confirmed as multi-drug-resistant (MDR). All the strains tested were susceptible to both lemongrass and citral with zones of inhibition varying between 17 to 80 mm. The mean minimum inhibitory concentration (MIC) and minimum bactericidal concentration (MBC) of citral (*mic*—0.14 % and *mbc*—0.3 % *v*/*v*) was lower than that of Lemongrass (*mic*—0.65 % and *mbc*—1.1 % *v*/*v*) determined using the microtitre plate method. Cell viability using human dermal fibroblasts (HDF; 106-05a) was determined following exposure to both compounds and a control (Grapeseed oil) using the XTT assay and the IC_50_ determined at 0.095 % (*v*/*v*) for citral and 0.126 % (*v*/*v*) for lemongrass. Grapeseed oil had no effect on cell viability. Live cell imaging was performed using the LumaScope 500 imaging equipment and changes in HDF cell morphology such as necrotic features and shrinkage were observed. The ability of lemongrass essential oil (EO) and citral to inhibit and kill MDR *A. baumannii* highlights its potential for use in the management of drug-resistant infections; however, in vitro cytotoxicity does suggest further tests are needed before in vivo or ex vivo human exposure.

## Introduction

The global threat of antimicrobial resistance (AMR) and infections caused by AMR bacteria has raised the need for urgent therapeutic discoveries and improvement of existing infection control and antimicrobial practices. In recent years, the Gram-negative bacterium *Acinetobacter baumannii* has been identified as a resilient and resistant pathogen (Perez et al. 2007). Outbreaks in hospital and community settings caused by multi-drug-resistant, extensively drug-resistant and pan-drug-resistant *A. baumannii* have been reported worldwide. According to Vila and Pachon (2011), new approaches and antimicrobial agents are needed for the control of *A. baumannii* infections as the few existing treatments have not been successful in managing infections caused by *A. baumannii*.

The failure of existing antibiotics in managing infections caused by AMR organisms has increased interest in alternative treatments. This is demonstrated by the breadth of literature published in the area of natural antimicrobials especially the antimicrobial effects of plant based essential oils (EOs). The effects EOs are wide ranging and include antibacterial, antifungal, antibiofilm, antiparasitic, antioxidant, antiviral, anticancer and many other reported effects; however, the information on bioactivity and toxicity of essential oils is not as extensively studied. Despite this, the commercial use and applications of EOs continues to grow, e.g. EOs are used in household cleaning products, cosmetics, perfumery, insecticides, disinfectant wipes, food and in management of infections in animals.

There are between 400 and 500 commercially produced essential oils (Tisserand and Young 2013) and one EO with a growing reputation is Lemongrass EO of the *Cymbopogon* species. The antimicrobial effect of whole lemongrass EO has been shown in previous studies with a wide range of in vitro activity including effects against AMR pathogens (Doran et al. 2009; Warnke et al. 2009; Adukwu et al. 2012). The strong antimicrobial activity of lemongrass has been attributed to a high citral content (Marongiu et al. 2006; Adukwu et al. 2012; Kumar et al. 2013; Kpoviessi et al. 2014). Both lemongrass EO and citral are generally regarded as safe (GRAS) for use as flavouring substances and is also an approved compound for use as a food additive and for human consumption (Food and drug Administration 2015a; 2015b).

Generally, cytotoxic activity of EOs and components on human cell lines have been studied with a larger proportion of these studies focusing on the effects of tea tree oil (Söderberg et al. 1996; Lis-Balchin et al. 2000; Hammer et al. 2006; Loughlin et al. 2008; Nielsen 2008). Kpoviessi et al. (2014) investigated the cytotoxic activity of lemongrass EO from four *Cymbopogon* species; *C.citratus*, *C. giganteus*, *C. nardus* and *C. schoenantus* against a human non-cancer diploid fibroblast cell line (W138) showing moderate toxicity of *C. citratus* against this W138 cell line. The cytotoxic effect of *C. flexuosus*, the EO in focus in our study and known to possess antimicrobial activity, was not determined in the Kpoviessi study, and the effect of the oil on dermal fibroblast is yet to be demonstrated to our knowledge. Citral on the other hand has been reported to cause several adverse reactions such as sensitisation and allergic contact dermatitis (Tisserand and Balacs 1995; Heydorn et al. 2003).

The focus of this study was to investigate the antimicrobial activity of *C. flexuosus* and citral against *A. baumannii* and to determine the cytotoxic activity of both *C. flexuosus* and citral on human fibroblasts which is important due to the growing usage of EOs in household applications and cosmetics as well as proposed usage in health care applications.

## Materials and methods

### Bacterial strains

Five *A. baumannii* strains from the University of the West of England, Bristol, UK, microbiology culture collection were used in this study. These were ATCC® BAA-1709™ (human isolate), ATCC® BAA-1710™ (human isolate), NCTC 12156 (ATCC 19606; type strain), ATCC 17978 (lung infection model; human isolate) and SM 37212, a clinical isolate obtained from the Pathology department at Southmead Hospital, Bristol, UK. The strains were maintained on brain heart infusion (BHI) agar (CM1136; Oxoid Ltd, Basingstoke, UK) and sub-cultured on a weekly basis. For inoculum preparation, single colonies were picked from a BHI agar plate into BHI broth (CM1135; Oxoid Ltd., Basingstoke, UK) and incubated overnight at 37 °C.

### EO and component

The lemongrass EO (*C. flexuosus*) used in this study was donated by Amphora Aromatics, Bristol, UK, whilst citral (95 %; synonym—3,7-dimethyl-2,6-octadienal, geranial and neral mixture; CAS Number 5392–40-5) was purchased from Sigma-Aldrich, Dorset, UK. The EO and citral were stored in a cool dark place and the containers kept tightly closed in a dry and well-ventilated place according to the safety data information.

### Susceptibility testing

The disk diffusion assay was used to determine antimicrobial susceptibility of the *A. baumannii* to a selection of antibiotics (Table 1) using the British Society for Antimicrobial Chemotherapy guidelines, version 13 (2014) and to whole lemongrass EO and citral. Using the agar overlay assay, bacterial lawns were prepared with the inoculum size adjusted to approximately 1.5 × 10^8^ CFU/ml. Ten microliters of lemongrass EO and citral were deposited onto 6-mm filter paper discs before placing them on the surface of Iso-sensitest agar (Oxoid; Basingstoke, UK). The agar plates were then incubated at 37 °C for 24 h and the diameter of the zone of inhibition (ZOI) measured in millimetres using a Vernier calliper. Each experiment was performed in triplicate. The controls were bacterial cultures without treatment.

**Table 1:** Inhibition zones (mm) of *A. baumannii* strains after 24 h exposure to selected antibiotics following BSAC guidelines (version 13, June 2014)

Antibiotic	Disc content (μg)	NCTC 12156 (ATCC 19606)	ATCC 17978	SM 37212	ATCC® BAA-1709™	ATCC® BAA-1710™
Ciprofloxacin	1	18.30 (R)	17.6 (R)	0.00 (R)	30.10 (S)	0.00 (R)
Gentamicin	10	20.70 (S)	19.6 (R)	7.42 (R)	31.70 (S)	7.10 (R)
Meropenem	10	26.50 (S)	27.7 (R)	21.00 (R)	36.80 (S)	25.70 (S)
Piperacillin/Tazobactam	75/10	24.50 (S)	25.00 (S)	18.90 (R)	86.00 (S)	20.00 (I)

Criteria for defining MDR, XDR and PDR in *Acinetobacter* spp. (Magiorakos et al. 2012); MDR: non-susceptible to ≥1 agent in ≥3 antimicrobial categories; XDR: non-susceptible to ≥1 agent in all but ≤2 categories; PDR: non-susceptible to all antimicrobial agents listed

(*R*) resistant, (*S*) sensitive, (*I*) intermediate

### Minimum inhibitory concentration and minimum bactericidal concentration

The method used in this study for determination of inhibitory and bactericidal activity of both lemongrass EO and citral was similar to that use in Adukwu et al. (2012) with similar concentration ranges 0.03, 0.06, 0.12, 0.5, 1, 2 and 4 % (*v*/*v*), and the only difference was the microplate reader. In this study, we used the Tecan Infinite 200 PRO, Reading, UK for optical density measurements. For minimum bactericidal concentration (MBC) determination, 10 μl was taken from each well (treated and untreated) after incubation and spot inoculated on BHI agar and incubated for 24 h at 37 °C. The concentration at which no growth was observed on subculture was determined as the MBC.

## Cytotoxic activity

### Cell cultures

Primary cell cultures of human dermal fibroblasts (HDF; 106-05a) were obtained from ECACC (European Collection of Cell Cultures, Porton Down, Sailsbury, UK). HDFs were cultured in DMEM (Sigma, Poole, UK, D6546) supplemented with 10 % (*v*/*v*) FBS (Sigma) and 1 % (*v*/*v*) GIBCO® GlutaMAX™ (ThermoFisher Scientific, Paisley, UK). Cells were maintained at 37 °C, in a humidified incubator at 5 % CO_2_ and passaged when 80 % confluent using 0.25 % Gibco® Trypsin-EDTA (ThermoFisher Scientific, Paisley, UK).

### In vitro cytotoxicity testing

The XTT assay (X4626 SIGMA, Poole, UK) was used in this study to assess cell viability. The HDFs were plated at 1 × 10^4^ cells/100 μl in sterile 96-well tissue culture microtitre plates and incubated in a humidified atmosphere of 5 % CO_2_ at 37 °C for 48 h to allow the cells to reach approximately 70 % confluence. The growth medium was discarded and replaced with growth medium containing serial dilutions of lemongrass EO or citral and further incubated for 1 h.

Cell viability was determined using a combination of the tetrazolium compound 2,3-Bis-(2-Methoxy-4-Nitro-5-Sulfophenyl)-2H-Tetrazolium-5-Carboxanilide (XTT, Sigma) and the electron coupling reagent phenazine methosulphate (PMS, Sigma). Each reagent was prepared separately by dissolving XTT at 1 mg/ml in pre-warmed (55 °C) media and dissolving PMS at 1.53 mg/ml in PBS. To 1 ml of the XTT solution, 5 μl of the PMS stock was added and then 0.25 ml of the XTT:PMS stock was added to 1-ml media to prepare the working XTT:PMS solution.

Following incubation for 1 h with lemongrass EO or citral, the medium containing the compounds was discarded and the cells were washed twice with PBS before incubation with 100 μl of the working XTT:PMS solution. Cells were incubated in the XTT:PMS reagent for 4 h and the absorbance read at 490 and 690 nm on a microtitre plate reader (BMG FLUOstar Omega). Each experiment was performed in quadruplicate on three separate occasions.

Controls included wells containing medium alone without cells (MA) and wells containing cells treated with medium alone without lemongrass EO or citral. For wavelength correction (corrects for optical imperfections), absorbance readings at 690 nm were first subtracted from the 490-nm readings. The data set was then blank corrected against the MA wells. The percentage viability was calculated as follows:

$$ \%\mathrm{viability}=\mathrm{mean}\ \mathrm{absorbance}\ \mathrm{of}\ \mathrm{treated}\ \mathrm{wells}\times 100/\mathrm{mean}\ \mathrm{absorbance}\ \mathrm{of}\ \mathrm{cells}\ \mathrm{without}\ \mathrm{treatment} $$

### Data analysis and statistics

The cytotoxicity expressed as IC_50_ (the concentration that caused 50 % cell death) was calculated by a four-parameter non-linear regression using GraphPad Prism software by plotting the percentage viability against the log of the oil concentration. The *t* test was used to compare the means of both test compounds whilst a linear regression analysis was used to correlate the cytotoxic activity of the major component (citral) to the lemongrass EO.

### Live cell imaging

HDF cells were imaged using the LumaScope 500 (Etaluma; Labtech, East Sussex, UK), which allows live cell imaging within a standard incubator. A ×40 objective lens was used under phase contrast settings. Images were acquired every minute for 120 min.

### Gas chromatography mass spectroscopy

Chemical analysis of the lemongrass and grapeseed EO was performed using the Agilent 6890 N Gas Chromatograph system (Agilent Technologies, USA) instrument with a HP-5 column (0.25 mm × 30 m × 0.25 μm) and an Agilent Technologies 5973 inert MS detector with MSD. Column: Agilent 190,915–433 capillary, 0.25 mm × 30 m × 0.25 μm. Capillary: 30 m × 250 μm × 0.25 μm nominal. The oven temperature program was the following: initial temperature of 50 °C, increasing by 15 °C/min to 240 °C, and held for 30 min. The samples were dissolved in hexane, and helium was used as the carrier gas with a 1 μl injector volume, an injector temperature of 300 °C and a split ratio 20:1. The injector and detector temperature were held at 280 °C. The resulting compounds were identified by comparing retention times and mass spectra with those of standards or their retention indices (RI) with published data and their mass spectra with the National Institute of Standards and Technology (NIST) library.

### Statistical analysis

Statistical analysis was conducted using GraphPad Prism 6. Significance levels were set at *P* = 0.05. Where the means of two samples were compared, the two-tailed *t* test was used and where the mean from more than two samples were compared, the one way analysis of variance (ANOVA) was applied.

## Results

### Antimicrobial studies

The *A. baumannii* isolates were screened for antimicrobial susceptibility following exposure to antibiotics from four different antibiotic groups. The results showed that the ATCC® BAA-1709™ was the most sensitive of the isolates, followed by the NCTC 12156 (ATCC 19606), the ATCC® BAA-1710™, ATCC 17978 and the SM 37212 in that order. The ATCC 17978 was resistant to three of the four antibiotics and was only sensitive to the Piperacillin/Tazobactam combination whilst the SM 32712 was resistant to all the antibiotics tested Ciprofloxacin (Quinolones), Gentamicin (aminoglycosides), Meropenem (Carbapenem) and Piperacillin/Tazobactam (Beta-lactam/beta-lactamase inhibitor). Both the ATCC 17978 and the SM 37212 fit the criteria for MDR, i.e. non-susceptibility to ≥1 agent and in ≥3 antimicrobial categories (Magiorakos et al. 2012).

**Fig. 1:**
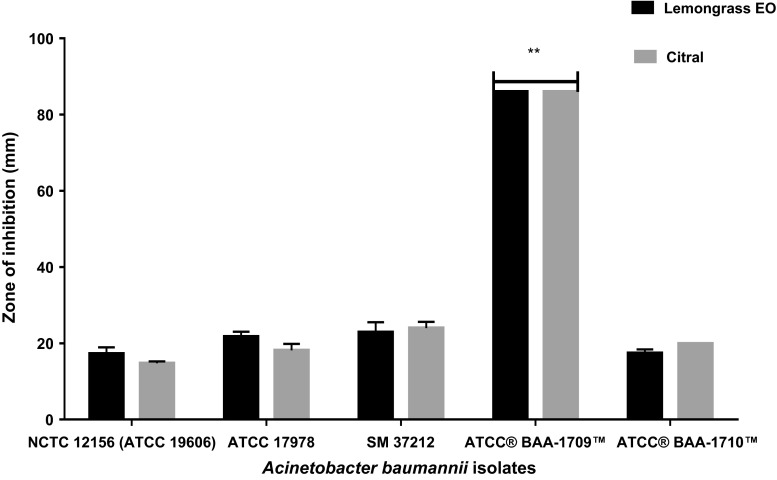
Comparison of the inhibition zones (mm) of whole lemongrass EO (*black bars*) and citral (*grey bars*) against A. baumannii isolates measured on agar medium. **The disk diffusion of ATCC® BAA-1709™ is significantly different from the other isolates (*P* = 0.02). When the disk diffusion of the other four isolates were compared, there was no significant difference (*P* = 0.25)

Disk diffusion assay results showed similar activity between whole lemongrass EO and citral (Fig. 1) although the activity of citral was slightly higher than that of whole lemongrass EO for three of the *A. baumannii* isolates (NCTC 12156, ATCC 17978 and SM 37212). The effect of both test compounds was significantly greater against the human isolate ATCC® BAA-1709™ (*P* = 0.02). This isolate was the most susceptible to the effects of the EO with full clearance of this organism on the agar plates following incubation, i.e. ZOI ≥ 86 mm. The other isolates demonstrated reduced susceptibility to both whole lemongrass EO and citral with inhibition zones between 15 and 28 mm for lemongrass EO and between approximately 22 and 27 mm for citral for the other tested strains.

The inhibitory and bactericidal activity of the EO and citral were determined using the broth micro dilution assay (Table 2). The minimum inhibitory concentration (MIC) for lemongrass was observed at a range between 0.25 and 1 % (*v*/*v*) whilst that for citral was between 0.06 and 0.25 % (*v*/*v*). The observed MIC was at a lower concentration when the organisms were exposed to citral then when exposed to the whole lemongrass EO. The MBC for lemongrass EO was between 0.5 and 2 % (*v*/*v*) for lemongrass EO and between 0.25 and 0.5 % (*v*/*v*) for citral. The highest combined MIC/MBC (least susceptible) was observed with the ATCC 1710 strain with MIC at 1 % (*v*/*v*) and MBC 2 % (*v*/*v*) when exposed to lemongrass EO although this was different with citral with the combined MIC/MBC of the least susceptible stain (NCTC 12156) at 0.25 % (*v*/*v*) and 0.5 % (*v*/*v*) for MIC and MBC, respectively (Table 2).

**Table 2:** Mean Inhibitory and bactericidal activity (% *v*/*v*) of whole lemongrass EO and citral against *A. baumannii* and *S.aureus* isolates. Each experiment was repeated in triplicate on three separate occasions

Microorganisms	Lemongrass EO (*C. flexuosus*)		Citral	
*A. baumannii sp.*	MIC	MBC	MIC	MBC
NCTC 12156	0.5	0.5	0.25	0.5
ATCC 17978	0.25	1	0.13	0.25
SM 37212	1	1	0.13	0.25
ATCC 1709	0.5	1	0.06	0.25
ATCC 1710	1	2	0.13	0.25
Mean	0.65	1.1	0.14	0.3
*S. aureus sp*.^a^				
HA-MSSA isolate	0.06	0.13	0.02	0.06
CA-MSSA (PVL + ve)	0.06	0.13	0.03	0.06
MSSA NCTC 13297	0.06	0.13	0.03	0.06
MRSA MW2	0.06	0.13	0.03	0.06
CA-MRSA (PVL + ve)	0.06	0.13	0.03	0.06
Mean	0.06	0.13	0.028	0.06

^a^Data for *S .aureus* from Adukwu (2013)

In all strains, the MIC determined following exposure to citral was lower than that for the whole lemongrass EO and with the exception of the NCTC 12156 strain with the MBC at 0.5 % (*v*/*v*), the MBC for the four other strains were lower than that of the lemongrass EO (Table 2).

## Cytotoxic activity

### XTT assay

**Fig. 2:**
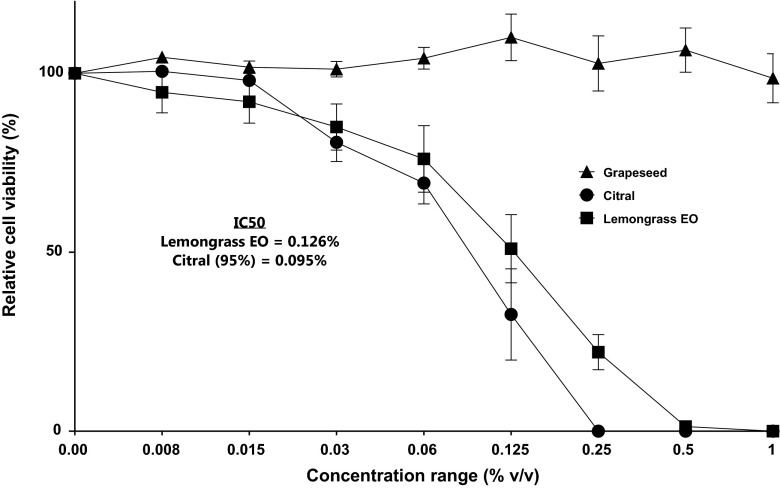
Dose-dependent cytotoxicity calculated as the relative cell viability (%) of whole lemongrass EO, citral and grapeseed EO (carrier oil) on HDF as determined by the XTT assay. Each experiment was performed in quadruplicate on four separate occasions. Grapeseed oil was also performed in quadruplicate and repeated on two separate occasions

Following exposure of the HDF cells to varying concentrations of lemongrass EO, cell viability was maintained between 80 and 100 % at 0.06 % (*v*/*v*) and the lower dilutions (Fig. 2). At 0.125 % (*v*/*v*), cell viability was approximately 60 % and at 2× the concentration, i.e. 0.25 % (*v*/*v*) the cell viability was approximately 25 %. At the tested concentrations higher than 0.25 % (*v*/*v*), exposure to lemongrass EO reduced the viability of the HDF cells to approximately 5 %.

When the HDF cells were exposed to citral at the same concentration ranges as the lemongrass EO, at the lowest concentrations between 0.008 and 0.016 % (*v*/*v*), there was no effect on cell viability. At 0.03 % (*v*/*v*), the cell viability was maintained between 80 and 100 %; however, at 0.06 % (*v*/*v*) the cell viability was approximately 75 %. At 0.125 % (*v*/*v*), the cell viability was reduced to approximately 42 %. At the higher concentrations ≥0.25 % (*v*/*v*), no viable cells were observed (Fig. 2).

The IC_50_ value obtained for lemongrass EO with the HDF was approximately 0.13 % (*v*/*v*) which was slightly higher than the IC_50_ of citral at approximately 0.1 % (*v*/*v*) (Table 1). Using two-tailed *t* test (two-tailed), the means of both lemongrass EO and citral EO were compared and the difference was not significant (*P* = 0.8254; *R* ^2^ = 0.003132). Grapeseed oil (the carrier oil) had no cytotoxic effects on the dermal fibroblasts and was therefore statistically different to the effects of both Lemongrass EO and citral (one way ANOVA; *P* = 0.0107; R2 = 0.3147).

### Live imaging

**Fig. 3:**
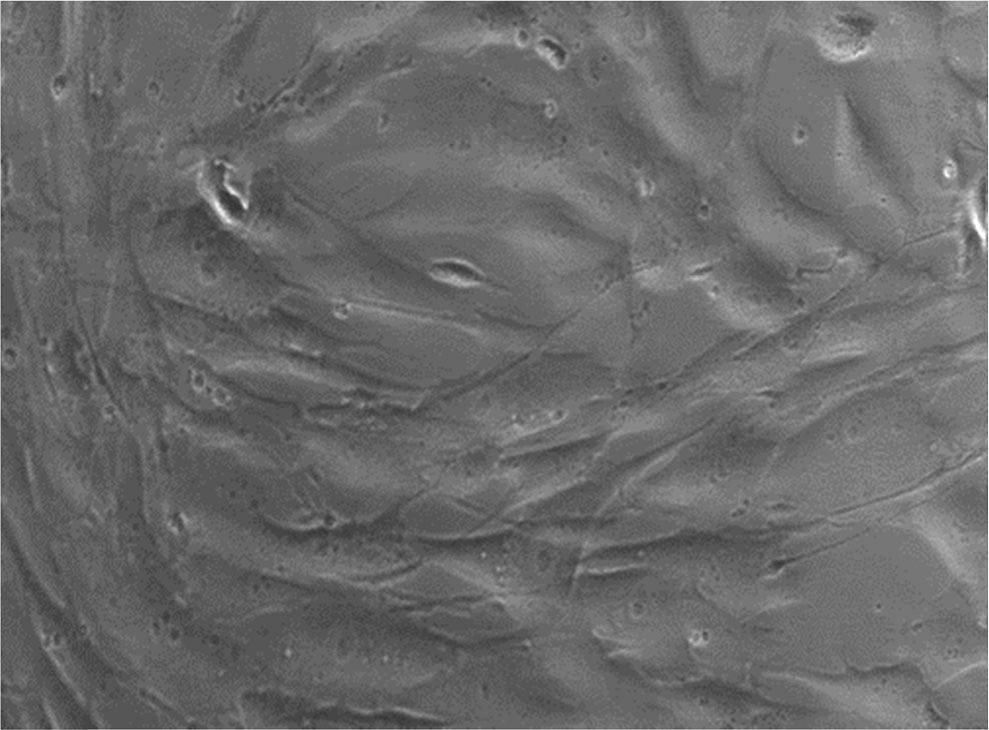
Human dermal fibroblasts (HDF; 106-05a) obtained from ECACC grown to 80 % confluence using the DMEM medium and supplemented with 10 % (*v*/*v*) FBS and 1 % (*v*/*v*) Gibco® GlutaMAX™ prior to treatment with test compounds. ×40 objective

**Fig. 4:**
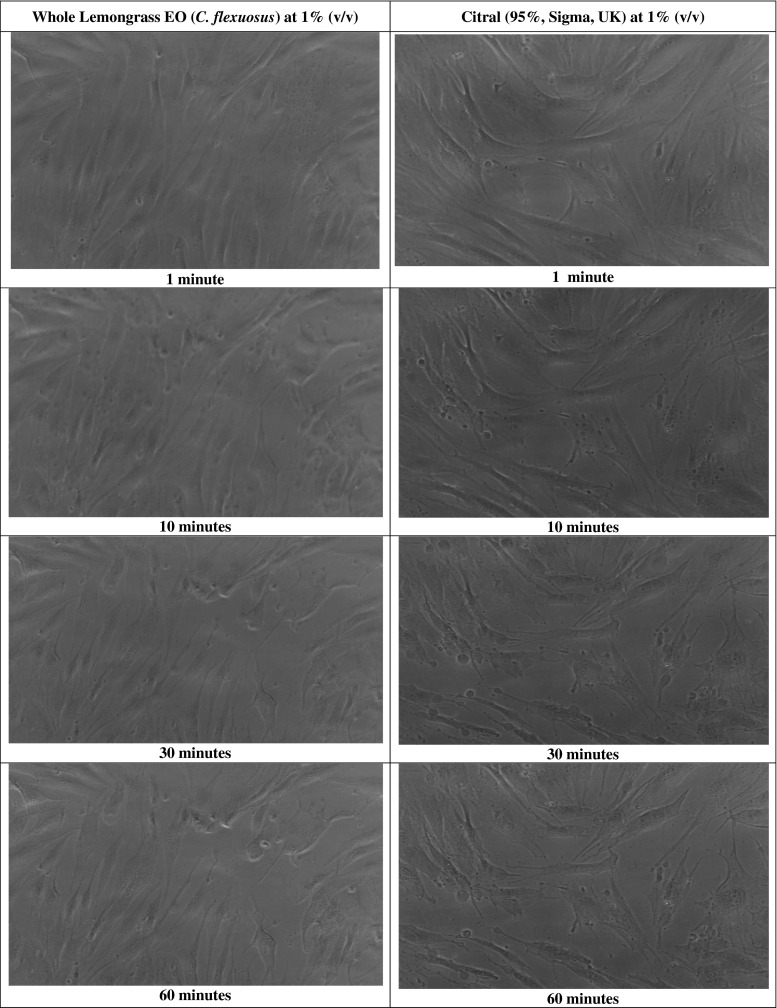
Cellular morphology changes of the HDF cells exposed to Lemongrass EO (C. flexuosus) at 1 % (*v*/*v*) and citral at 0.25 % (*v*/*v*) over a 2-h period. The images shown represent images captured at 1, 10, 30 and 60 min, respectively, using the LumaScope 500. ×40 objective

The HDF cells prior to exposure with lemongrass or citral are shown in Fig. 3. When the HDF cells were exposed to lemongrass EO at 1 % (*v*/*v*) or citral at 0.25 % (*v*/*v*), changes in cell morphology were observed during the 120-min exposure. Within 5 min of citral exposure, the HDFs showed no noticeable changes; however, following 10 min, there was rounding up of cells. Following 20 min, there was a noticeable increase in the rounding up of cells and subsequent visible necrosis and cell death within 60 min (Fig. 4). Similarly, lemongrass EO also induced the rounding up of the cells following 10 min, with an increase following 20 min. Necrosis and presence of loosely attached cells was observed at 30, 60 and 120 min (Fig. 4).

## Discussion

*A*. *baumannii* has been described as AMR bacterium, and in this study, the isolates and type strain investigated for antimicrobial susceptibility to antibiotics demonstrated a range of susceptibilities to the different antibiotic groups tested (Table. 1). In 4/5 *A. baumannii* tested in this study, antibiotic resistance was identified and we observed varying resistance characteristics based on the criteria defined by Magiorakos et al. (2012). This included MDR in the ATCC 17978, PDR in the clinical isolate SM 37212 and possibly XDR in the ATCC BAA-1710™ isolate; however, further screening will confirm if this is a MDR or an XDR isolate. Following antibiotic screening, the isolates were exposed to lemongrass EO and major component (citral—approximately 90 % determined by GC-MS; Table 3) and found to be susceptible, including the isolates identified as MDR.

**Table 3:** GC-MS Analysis of lemon grass and Grapeseed EOs showing major components

Component	Lemongrass EO	Grapeseed EO
Geraniol	8.235	–
Linalool	2.765	–
Neral^a^	38.491	–
Geranial^a^	50.509	–
1,2,benzenedicarboxylicacid, mono1,2,ethylhexyl ester	–	77.131
Diocxylpthalate	–	22.869
Percentage of total	100	100

^a^Neral and Geranial are isomers of citral hence when combined approximately 89 % in this study

We observed that the human isolate ATCC BAA-1709™ was most sensitive to the antibiotics tested in this study and subsequently the most sensitive to the effects of both lemongrass EO and citral. Although it is difficult to make direct comparisons between the activity of the antibiotics and the EO/citral, the results suggest some similarity in level of activity between the different compounds. Other studies that have investigated activity of EOs and antibiotics have predominantly focused on the synergistic action (Rosato et al. 2007; Duarte et al. 2012; Aleksic et al. 2014 **)** rather than any comparisons between both compound types. However, there is a need for further research into how the effects of lemongrass EO and/or citral relate to the effect of antibiotics on drug sensitive and resistant bacteria. The MIC and MBC results suggest that the effect of citral is greater than that of whole lemongrass EO. This was observed previously in Adukwu et al. (2012) where the inhibitory and bactericidal concentrations of citral were also lower against different isolates of *S.aureus* further indicating that pure citral is more potent than whole lemongrass EO against different bacteria species.

Following determination of antimicrobial activity, we analysed the effect of the EO/citral on dermal fibroblasts.

In the study by Kpoviessi et al. (2014), the authors found that only *C. citratus* demonstrated a moderately toxic activity against the W138 cells (IC_50_ = 39.77 μg/ml) whilst the other *Cymbopogon* species had low cytotoxicity against same fibroblast cells and suggested the need for further toxicity studies. The authors also found that citral, approximately 75 % from GC-MS analysis in their study, also demonstrated a moderately toxic activity (IC_50_ = 39.48 μg/ml).

In our study, following exposure of the dermal fibroblast cells to lemongrass EO at the concentration 0.125 % (*v*/*v*), cell viability was reduced to approximately 60 %, and at 0.25 (*v*/*v*), cell viability was reduced to approximately 25 %. At 0.125 % (*v*/*v*), the effect of citral on cell viability was approximately 15–16 % greater than the activity of whole lemongrass EO on the fibroblast cells, and at 0.25 % (*v*/*v*), there was total loss in cell viability at the 1-h exposure time. There was no loss in cell viability at ≤0.03 % for lemongrass EO although following exposure to citral at the same concentration, there was approximately a 15 % loss in cell viability. Overall, comparing the effect of both whole lemongrass EO and citral the observation is that the difference in cytotoxic activity at the concentrations tested is approximately 20 % thus, suggesting that as the concentration is increased, the cytotoxicity of citral increases in favour of citral.

This is not the first time that the cytotoxic effects of EO components has been observed to be greater than the whole oil. In Hammer et al. (2006), the components of TTO were shown to be more cytotoxic against human cells lines in comparison to whole TTO. A similar response was found in the study by Prashar et al. (2004) which showed that both linalool and linalyl acetate (major components in lavender accounted for approximately 85 % of the oil) were more cytotoxic than the whole EO. In contrast, Hayes and Markovic (2002) showed similar cytotoxic action (similar IC_50_ results) and no significant difference when citral was compared with the Australian lemon myrtle EO. This is similar to the findings in our study as there was no significant difference between the IC_50_ of whole lemongrass EO and citral (*P* = 0.8254) and although the oils are different, the concentration of citral in our study (>89 % *v*/*v*) from GC-MS analysis (Table 3) was similar to the citral content in the Hayes and Markovic study (>92 % *v*/*v*).

Recommendations by the Nomenclature Committee on Cell Death (NCCD) are that at least two distinct methods of assessments should be used for cell death analysis (Kroemer et al. 2009), and in a follow-up paper by Galluzzi et al. (2009) on guidelines for use and interpretation of cell death assays, using methods such as enzymatic assays which involve the incorporation of exclusive dyes and long-term survival assays could be fundamental in answering the questions surrounding cell death as described by the NCCD. In this study, we used the XTT assay, now a common tool used in cell viability assays (Berridge et al. 2005) for determining the effect of the test compounds on cell survival and the live cell imaging for morphological examinations of the treated HDF.

Morphological examinations of the treated HDF showed that both lemongrass EO (1 % *v*/*v*) and citral (0.25 % *v*/*v*) caused damage to the fibroblasts within the first few minutes and total cell death within the 120-min exposure time at the tested concentrations. Close examination of the fibroblasts treated by lemongrass EO showed possible cell damage with features such as rounding up of the cells, cell shrinkage and retraction of the pseudopods suggesting damage as a result of apoptosis. The citral-treated fibroblasts showed an increased amount of blebbing (spherical membrane protrusions), cell retraction and shrinkage suggesting apoptosis as well as necrotic features on the damaged fibroblasts (Leverrier and Ridley 2001; Charras and Paluch 2008). The morphological changes and damage observed at the tested concentrations support the cell viability results which suggest lack of metabolic activity linked to the inhibition of cell proliferation thus cell damage and cell death features. To support the morphological observations, future studies would benefit from assessments to determine apoptotic and/or necrotic activity such as caspase activation and nuclear fragmentation assays as recommended by the NCCD (Kroemer et al. 2009) Also, further tests on cytotoxicity using this species of lemongrass in vitro and in vivo is needed on different cell lines before deciding whether or not to use this EO for management of human topical infections.

In summary, we have shown that lemongrass EO and citral were effective at inhibiting and killing MDR *A. baumannii*. This enhances the potential of the lemongrass EOs as a possible alternative to antibiotics owing to its ability to effectively inhibit and kill both MDR Gram-positive and Gram-negative bacteria. Our study also demonstrate that at concentrations shown to be bactericidal against Gram-negative drug-resistant *A. baumannii*, whole lemongrass EO and citral act against dermal fibroblast cells in vitro. However, at concentrations where citral and whole lemongrass EO were previously shown to be inhibitory and bactericidal against drug-resistant MRSA and MSSA (Adukwu 2013, PhD thesis), cell viability of the HDF cells remain high (≥70 %) which is a positive finding as it offers the potential for of these use in managing contamination in both clinical and non-clinical settings where drug-resistant staphylococcal infections remains a major problem globally.
